# Low risk of hepatotoxicity from rifampicin when used for cholestatic pruritus: a cross‐disease cohort study

**DOI:** 10.1111/apt.14579

**Published:** 2018-02-22

**Authors:** G. J. Webb, S. R. Rahman, C. Levy, G. M. Hirschfield

**Affiliations:** ^1^ Birmingham Biomedical Research Centre (BRC) National Institute for Health Research (NIHR) University of Birmingham Birmingham UK; ^2^ University of Miami Miami FL USA; ^3^ Institute of Translational Medicine Birmingham Health Partners University Hospitals Birmingham NHS Trust Birmingham UK

## Abstract

**Background:**

The use of rifampicin for cholestatic pruritus is accompanied by concerns over safety, but the availability of real‐world prescribing data is relatively limited.

**Aim:**

We sought to describe the rate and characteristics of rifampicin‐induced hepatitis in a mixed aetiology cohort of patients with established liver disease and cholestatic pruritus.

**Methods:**

Retrospective review of records for out‐patients commenced on rifampicin for pruritus 2012‐2016 inclusive. Rifampicin‐induced hepatitis was recorded where alanine aminotransferase activity (ALT) increased to both ≥5 × baseline and ≥5 × upper limit of normal (ULN), or to both ≥3 × baseline and ≥3 × ULN with concurrent elevation in serum bilirubin to ≥2 × baseline and ≥2 × ULN, in addition to a Roussel‐Uclaf Causality Assessment Method score of “probable” or “highly probable” for rifampicin causality.

**Results:**

After exclusions, we reviewed 105 patients who took rifampicin for a median of 131 days. Most had primary biliary cholangitis or primary sclerosing cholangitis; 40 (38.1%) were men and median age was 44 years (IQR: 32‐57). 44 (41.9%) patients had baseline serum bilirubin ≥2 × ULN and 28 (26.7%) ALT ≥3 × ULN. 5 (4.8%) developed rifampicin‐induced hepatitis at a median of 70(range 27‐130) days after drug initiation. No individual or laboratory baseline characteristics were significantly associated with subsequent development of hepatitis. All cases of hepatitis recovered after drug cessation, although one patient was hospitalised and received corticosteroids.

**Conclusions:**

Given the efficacy of rifampicin for an important sub‐group of those with cholestatic pruritus, adult patients, including those with jaundice, can be counselled that 95% of prescriptions are safe, and where hepatitis occurs, including at long latency, drug cessation appears effective.

## 1 INTRODUCTION

Pruritus is a frequent and distressing complication of liver disease, especially cholestatic liver disease.^1^ The antimicrobial drug rifampicin (USAN: rifampin) is recognised as a therapeutic agent for pruritus having shown efficacy in the majority of controlled trials,^2^, ^3^, ^4^, ^5^, ^6^ and is currently recommended by major international guidelines in the therapy of pruritus in cholestatic liver disease.^7^, ^8^

Rifampicin has been reported to be associated with hepatitis in a minority of patients receiving the drug as therapy for mycobacterial infections.^9^, ^10^ This is especially the case where there is combination therapy with other potentially hepatotoxic agents^11^ and is associated with a variety of risk factors^12^ To date meta‐analyses of previous controlled studies of rifampicin for pruritus have concluded that the treatment is safe, with the largest study of 61 patients recording no clinically significant episodes of hepatitis.^13^, ^14^ However, despite these meta‐analyses, individual reports of rifampicin‐induced hepatitis during therapy for pruritus do exist including with occurrence after the short‐term follow‐up typically used in trials.^15^, ^16^ It remains uncertain as to how best to counsel patients being prescribed rifampicin off‐label for therapy of cholestatic pruritus given a lack of estimates regarding its potential to cause liver injury. This is especially the case in those with pre‐existing jaundice.

Given the immediate utility to prescribers, we have evaluated the occurrence of hepatitis in a cohort of 105 patients with established liver disease of mixed aetiology in whom rifampicin was prescribed for pruritus. In so doing, we provide real‐world data of use to all prescribers when counselling patients.

## 2 METHODS

With the permission of University Hospitals Birmingham, a retrospective review of cases notes of all patients attending University Hospitals Birmingham on an out‐patient basis from 2012 to 2016 inclusive and who received new prescriptions for rifampicin without concurrent isoniazid was conducted. Details of demographics, laboratory variables and clinical course were reviewed.

Cases were considered to represent rifampicin‐induced hepatitis if laboratory values met the criteria of the DILI Expert Working Group:^17^

1. either, a rise in serum aspartate aminotransferase (AST) or alanine aminotransferase (ALT) activity to both ≥5 × pre‐rifampicin baseline and ≥5 × upper limit of normal (ULN);
2. or, a rise in ALT or AST to both ≥3 × pre‐rifampicin baseline and ≥3 × ULN with a concurrent rise in serum bilirubin to both ≥2 × pre‐rifampicin baseline and ≥2 × ULN;
3. and, a Roussel‐Uclaf Causality Assessment Method (RUCAM) score of ≥6 (“Probable” or “Highly probable”) for rifampicin hepatitis.^18^

Comparative statistics were used to compare groups that did, and that did not, develop hepatitis whilst taking rifampicin: the chi‐squared test was used to compare categorical variables whilst the Mann–Whitney U‐test was used to compare non‐normally distributed numeric variables with normality assessed using the Shapiro‐Wilk test; a *P* value of < 0.05 was considered significant. Analyses were performed with StataMP v15.0 (StataCorp, College Station, TX, USA)

## 3 RESULTS

**Figure 1:**
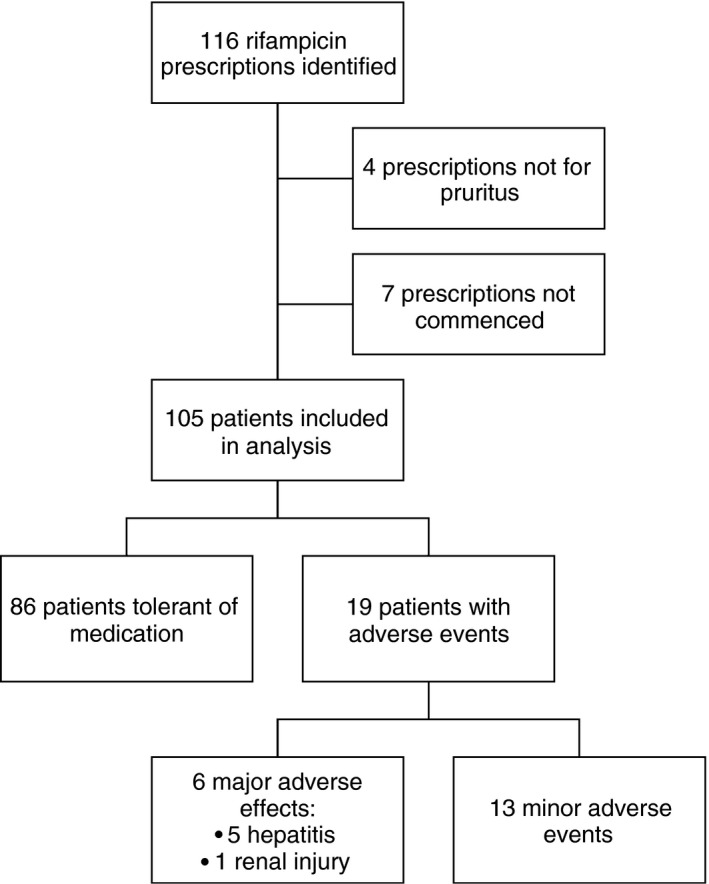
Patient selection. Patient cohort selection and exclusions from all patients prescribed rifampicin without isoniazid by the department of liver medicine from 2012 to 2016 inclusive

We identified 116 out‐patients prescribed rifampicin without concurrent isoniazid by the department of liver medicine between 2012 and 2016 inclusive (Figure 1). Of these, four (2.4%) prescriptions were not made for the treatment pruritus and seven (6.0%) prescriptions were never commenced. 105 patients were therefore included in the final analysis.

One thousand three hundred and eighteen patients were prescribed rifampicin without isoniazid by departments other than liver medicine over the same time period. To assess whether these patients had received rifampicin for pruritus, 116 records were randomly selected. None had received rifampicin for pruritus: 45 (38.8%) received rifampicin for skin and wound infections; 22 (19.0%) for mycobacterium tuberculosis infections; 17 (14.7%) for bone or joint infections; 17 (14.7%) as anti‐microbial prophylaxis; 8 (6.9%) for nontuberculosis mycobacterial infections; and 7 (6.0%) for other infections. Based on the absence of prescriptions of rifampicin for pruritus by departments other than liver medicine, only prescriptions by the department of liver medicine were further evaluated.

The characteristics of the patient cohort are recorded as Table 1. Of the 105 patients analysed, 94 (89.5%) were already prescribed medications other than rifampicin at the point of rifampicin prescription, with a median of 5 (IQR 3‐6) nonrifampicin medications. Major concurrent medications were 66 (62.9%) taking ursodeoxycholic acid, 33 (31.4%) proton pump inhibitors, 26 (24.8%) cholestyramine, 22 (21.0%) nonrifampicin antibiotics, 21 (20.0%) 5‐aminosalicylic acid preparations, 15 (14.3%) anti‐histamines, 9 (8.6%) azathioprine or 6‐mercaptopurine, 10 (9.5%) prednisolone and 8 (7.6%) statins. Two (1.9%) patients were prescribed concurrent sertraline and one patient (1.0%) was prescribed naltrexone.

**Table 1:** Baseline characteristics of patient cohort

Variable	Hepatitis (5)	No hepatitis (100)	*P*
Gender (n)			
Female	3 (60%)	62 (62.0%)	1.000
Male	2 (40%)	38 (38.0%)	
Age (years + IQR)	37 (28‐43)	44 (32‐59)	0.257
BMI (kg/m² + IQR)	25.6 (25.5‐25.9)	24.3 (21.8‐27.1)	0.309
Nonrifampicin medications (n + IQR)	5 (3‐6)	4 (3‐6)	0.294
Starting rifampicin dose (mg/d)			
150	0 (0.0%)	19 (19.0%)	0.479
300	5 (100%)	77 (77.0%)	
600	0 (0.0%)	4 (4.0%)	
Baseline laboratory values			
ALT (IU/mL + IQR)	86 (85‐95)	77 (41‐152)	0.500
AST (IU/mL + IQR)	123 (72‐191)	85 (23‐131)	0.300
Bili (μmol/L + IQR)	29 (17‐99)	36 (14‐87)	0.630
ALP (IU/mL + IQR)	399 (272‐414)	396 (221‐674)	0.690
Alb (g/dL + IQR)	41 (41‐44)	42 (36‐45)	0.880
Primary liver diagnosis (n)			
PSC	1 (20.0%)	43 (43.0%) [1]	0.900
PBC	3 (60.0%)	33 (33.0%) [3]	
Congenital	—	4 (4.0%)	
DILI	1 (20.0%)	3 (3.0%)	
Ischaemic cholangiopathy	—	3 (3.0%) [1]	
Other	—	3 (3.0%)	
ALD	—	2 (2.0%) [1]	
IgG4	—	2 (2.0%) [1]	
SSC	—	2 (2.0%)	
Transporter deficiencies	—	2 (2.0%)	
Cancer	—	1 (1.0%)	
Hepatitis C	—	1 (1.0%)	
ICP	—	1 (1.0%)	
Documented alcohol excess (n)	1 (20.0%)	3 (3.0%)	0.053
HBsAg + (n)	—	—	
HCVAb + (n)	—	2 (2.0%)	—

Numbers in square brackets denote the number of patients who had previously undergone liver transplantation; *P* values are calculated by the Mann–Whitney U‐test or χ² test as appropriate.

BMI, body mass index; ALT, alanine aminotransferase; AST, aspartate aminotransferase; Bili, serum bilirubin; ALP, alkaline phosphatase; Alb, albumin; PSC, primary sclerosing cholangitis; PBC, primary biliary cholangitis; DILI, drug‐induced liver injury; ALD, alcohol‐induced liver disease; IgG4, IgG4‐related disease; SSC, secondary sclerosing cholangitis; ICP, intrahepatic cholestasis of pregnancy.

At baseline, many patients had significantly deranged liver biochemistry (Table 1). 44 (41.9%) patients had a baseline bilirubin >2 × ULN, 23 (21.9%) patients had a baseline bilirubin ≥ 100 μmol/L (≥ 5.8 mg/dL), and 11 (10.5%) had a baseline ALT >5 × ULN with 28 (26.7%) >3 × ULN.

**Figure 2:**
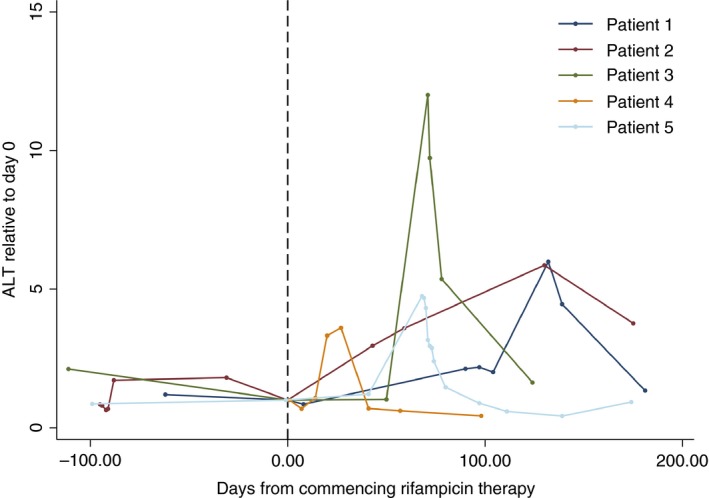
Course of serum alanine aminotransferase activity measurements in the five patients who developed rifampicin‐associated liver injury. Each dot represents a test. Values are normalised to the value recorded on the day of drug initiation. Patient numbers correspond to those presented in Table 2

Of the 105 patients followed up for a median of 131 days (IQR 52‐295) taking rifampicin, 5 (4.8%) patients diagnosed with hepatitis consistent with rifampicin‐induced hepatitis with a RUCAM score ≥ 6 (Figure 2; Table 2). The median time to diagnosis of rifampicin‐induced hepatitis was 70 days (range 27‐130). There were no significant differences in baseline characteristics between patients who were diagnosed with rifampicin‐induced hepatitis and those that were not (Table 1). One further patient was diagnosed with rifampicin‐associated acute kidney injury without associated hepatitis.

**Table 2:** Liver biochemistry at baseline and at diagnosis of rifampicin induced hepatitis

Patient			1	2	3^a^	4	5^b^
Major diagnosis			PBC	PSC	PBC	DILI	PBC
Age (y)			55	28	37	26	43
Gender (M/F)			M	M	F	F	F
Nonrifampicin prescriptions			6	3	6	3	4
RUCAM score			8	7	11	8	6

Variable	Normal range	Units	Laboratory values at baseline				
Bili	<22	(μmol/L)	99	17	15	348	29
ALP	35‐105 (F)/40‐130 (M)	(IU/L)	652	399	178	414	272
AST	5‐43	(IU/L)	168	—	65	78	213
ALT	5‐41	(IU/L)	85	95	41	86	221
Alb	34‐51	(g/L)	37	41	44	41	45
Cr	50‐111	(μmol/L)	71	71	63	61	59
eGFR	—	(mL/min)	>90	>90	>90	>90	>90
INR	0.8‐1.2	—	1.0	0.9	1.1	1.0	1.0
Eosinophils	<0.4	×10^9^/L	0.1	0.2	0.2	0.1	0.1
Time until diagnosis^b^	—	(d)	97	130	70 (14)	27 (20)	70

Variable	Normal range	Units	Laboratory values at diagnosis				
Bili	<22	(μmol/L)	201	39	126	72	186
ALP	35‐105 (F)/40‐130 (M)	(IU/L)	360	851	227	200	176
AST	5‐43	(IU/L)	286	—	646	158	—
ALT	5‐41	(IU/L)	186	557	492	310	1050
Alb	34‐51	(g/L)	34	39	43	44	33
Cr	50‐111	(μmol/L)	78	58	76	57	61
eGFR	—	(mL/min)	90	>90	74	>90	>90
INR	0.8‐1.2	—	1.7	1.1	1.3	1.0	1.9
Eosinophils	<0.4	×10^9^/L	0.1	0.2	0.3	0.2	0.1

RUCAM, Roussel‐Uclaf causality assessment method score where ≥ 6 represents probable drug (rifampicin)‐induced hepatitis; PBC, primary biliary cholangitis; PSC, primary sclerosing cholangitis; DILI, (cholestatic) drug‐induced liver injury. Bili, serum bilirubin (μmol/L); ALP, alkaline phosphatase (IU/mL); AST, aspartate aminotransferase (IU/mL); ALT, alanine aminotransferase (IU/mL); Alb, serum albumin (g/L); Cr, serum creatinine (μmol/L); INR, international normalised ratio.

Days from prescription to diagnosis, numbers in brackets indicate time from dose uptitration.

a Patient received a re‐challenge with rifampicin after resolution of initial hepatitis which again provoked significant elevations in serum transaminase activity.

b Patient admitted for treatment with corticosteroids.

During our median follow‐up of 809 days, we identified 29 patients who met biochemical criteria for potential DILI. Of these, 21 had undergone liver transplantation immediately prior to the derangement in liver biochemistry and were not further analysed. Of the remaining 8, 5 represented rifampicin‐related DILI as assessed by RUCAM and are described in Table 2. Three patients’ derangements in liver biochemistry were scored as not related to rifampicin. One patient reached biochemical criteria at 451 days following rifampicin initiation with a RUCAM score of −2 and a clinical course involving excess alcohol consumption; one at 545 days with a RUCAM score of −2 and a clinical diagnosis of deteriorating PSC; and one at 1239 days with a RUCAM score of −1 and with a clinical course that involved an undiagnosed febrile illness associated with cerebrospinal fluid lymphocytosis.

During the first 120 days of follow‐up, we assessed for milder fluctuations in liver biochemistry. Serum ALT activity rose to ≥2 × baseline and ≥2 × ULN in 6 (6.0%) of 101 patients not considered to have developed rifampicin induced liver injury (range 2.1‐3.8 fold rise). For serum bilirubin, 5 of 101 (5.0%) patients developed elevations to ≥2 × baseline and ≥2 × ULN without concurrent significant elevations in serum ALT activity (range 2.3‐3.0 fold rise).

**Figure 3:**
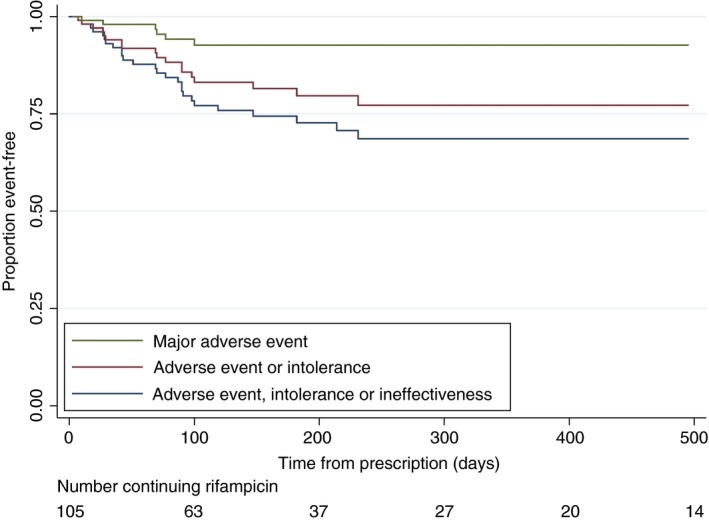
Proportion of patients event‐free after initial rifampicin prescription. Those who stopped taking rifampicin because of a major adverse event (hepatitis or renal failure) are shown in green; those who stopped because of an adverse event or intolerance are shown in red; those who stopped taking rifampicin because of an adverse event, intolerance or reported ineffectiveness are shown in blue. In all instances if the drug was stopped because of resolution of pruritus, liver transplantation, death or reasons other than an adverse event, intolerance or ineffectiveness, this was not recorded as a positive event at censoring

Other than the 6 patients who developed major adverse effects, 17 (16.2%) patients reported resolution of pruritus and stopped taking rifampicin, 9 (8.6%) reported a lack of efficacy despite dose uptitration, 13 patients (12.4%) reported intolerance (1 abdominal discomfort, 1 discolouration of bodily secretions, 5 gastro‐intestinal disturbance, 1 localised perineal skin reaction, 1 lethargy and 4 without a recorded reason). 21 (20.0%) received liver transplants, 3 (2.9%) were transferred to palliative care, 2 (1.9%) died, 5 (4.8%) stopped taking rifampicin for unrecorded reasons, and 29 (27.6%) continued to take rifampicin at the point of data collection. Figure 3 details the proportion of patients who remained free of major adverse events, minor adverse events and who continued to take rifampicin over the time period studied.

During treatment, 21 (20.0%) of patients received an uptitration in their dosage of rifampicin. Two of the five patients who developed rifampicin‐induced hepatitis received an uptitration in rifampicin dosage prior to developing hepatitis.

Of those five patients who developed rifampicin‐induced hepatitis, liver biochemistry resolved to baseline after cessation of rifampicin in four (80%). One patient who was treated with rifampicin for pruritus associated with primary biliary cholangitis was admitted, underwent a liver biopsy which showed bridging fibrosis with a patchy predominantly mononuclear infiltrate containing both lymphocytes and plasma cells, some interface hepatitis and marked‐and in places confluent‐parenchymal hepatocellular necrosis. She was treated with corticosteroids with subsequent resolution of liver biochemistry to baseline. The one patient with acute kidney injury recovered to baseline renal function after cessation of rifampicin.

## 4 DISCUSSION

Here, we present the largest, real‐world, cohort of patients treated with rifampicin for pruritus in liver disease published to date. Within our mixed aetiology cohort, we demonstrate that 95% of patients did not have any concern for hepatotoxicity, but that in 5% a rifampicin‐induced hepatitis was diagnosed. This finding is in contrast with the literature from controlled trials of rifampicin for pruritus where no cases of hepatitis were reported, but is consistent with isolated case reports and the literature derived from tuberculosis therapy. Our hepatitis rate of 4.8% is higher than the median of 1.1% reported for tuberculosis treatment regimens not containing isoniazid by one meta‐analysis, but is within the range of rates reported for tuberculosis patients treated with both isoniazid and rifampicin,^11^ and less than reported rates of 10%‐20% for any increase in serum transaminase activity reported with rifampicin therapy.^19^

Attributing liver injury to a given potential causative agent is challenging. In this study we used the established RUCAM, which has been widely used in other studies and has the benefit of being points‐based rather than explicitly relying on individual opinion. However, it is important to note that recent commentary and guidelines have also emphasised the value of expert opinion in diagnosing DILI.^20^, ^21^ Furthermore, the RUCAM has been suggested to potentially underestimate the rate of drug‐induced liver injury and to demonstrate more inter‐assessor variability than other methodology.^22^ In this study, we used a variation on the original RUCAM to account for deranged baseline liver biochemistry. Although such an adjustment is consistent with that promoted by a body expert opinion, it represents a variation from the initial RUCAM specification and is therefore likely to have differing sensitivity and sensitivity for diagnosing DILI.^17^

Although the number of patients who developed rifampicin‐induced hepatitis in our cohort is small and our analysis is retrospective, there were no statistically significant distinguishing features that predicted subsequent development of hepatitis; a much larger cohort would, however, be necessary to investigate risk factors more conclusively. This is in contrast with the work on those treated for mycobacterial infection with rifampicin, where alcohol excess, low BMI, low serum albumin, age and gender have been suggested as predisposing factors.^10^, ^11^, ^23^, ^24^ Multi‐centre studies will be needed to further investigate potential risk factors for rifampicin‐induced hepatitis in the context of liver disease.

Our cohort included some 23 patients with a baseline serum bilirubin of over 100 μmol/L ( ≥ 5.8 mg/dL). The UK package insert of rifampicin states that its use is contra‐indicated in jaundice, although anecdotally many liver clinicians will consider using rifampicin for the treatment of pruritus despite jaundice.^25^ We note, however, that it is our practice to co‐prescribe oral vitamin K for icteric patients receiving rifampicin to reduce the risk of coagulopathy.^26^ None of our markedly jaundiced patients developed hepatitis and we note that a separately reported group of markedly jaundiced patients with hepatocellular secretory failure predominantly attributed to biliary transporters dysfunction are reported as having safely received rifampicin for up to 10 weeks.^27^ Of our cohort, although one patient required corticosteroids and admission to hospital, none of our 105 patients developed life‐threatening complications from rifampicin despite some having advanced liver disease at baseline. Given that there was no established protocol for the monitoring of liver biochemistry during the time period assessed, it is possible that some further sub‐clinical rifampicin‐induced hepatitis may have escaped diagnosis. Nevertheless, our results suggest that rifampicin therapy is relatively safe in cholestatic jaundice.

Liver patients with cholestatic disorders may be prescribed multiple therapeutic compounds and our cohort was taking a median of 5 nonrifampicin medications. Although this number did not vary between groups that did and did not develop rifampicin‐associated hepatitis, we cannot exclude the potential for drug‐drug interactions promoting hepatitis, especially considering that over 20% of our patients were taking long‐term antibiotics, predominantly as prophylaxis against recurrent cholangitis. A further variable that cannot be accounted for within the sample size of this mixed aetiology cohort is interactions between rifampicin prescription and subtype of underlying cholestatic liver disease.

The median time to onset of hepatitis in our cohort was 70 days from initiation of rifampicin therapy. Most of the patients were followed up for longer than this period with the majority finding rifampicin tolerable and effective. Notably our median time of 70 days until onset of hepatitis is longer than the reported median interval to the development of hepatitis in cohorts of tuberculosis patients of approximately 15 to 30 days.^24^, ^28^ Importantly, two of our patients developed hepatitis after the end of the period of biochemical monitoring suggested by recent guidelines.^8^ We note that two of the three cases of rifampicin‐induced hepatitis reported by Prince et al occurred at 11 and 14 months after drug initiation.^15^ Again, given the absence of protocol liver biochemical monitoring and the retrospective nature of this study, it is possible that the number of cases of rifampicin‐induced hepatitis described here is an underestimate with cases meeting our criteria potentially being missed.

Our study provides real‐world experience of the use of rifampicin in cholestatic pruritus. We demonstrate useful reference data for clinicians who should counsel patients carefully: reassuringly 95% of our patients did not develop any liver injury, and the 5% that did develop hepatitis all recovered after drug cessation with only one patient requiring further intervention. Many patients with marked jaundice and advanced liver disease took rifampicin safely. In the absence of alternative safe, licensed and equally effective agents, clinicians may consider, the use of rifampicin in cholestatic pruritus.

## AUTHORSHIP

*Guarantor of the article*: Gwilym J Webb.

*Author contributions*: GJW, CL and GMH conceived the study. GJW and SRR collected the data. All authors then contributed to data analysis and drafting the manuscript.
